# Retrospection

**Published:** 1883-01

**Authors:** J. B. Chandler


### RETROSPECTION.

J. B. CHANDLER.

Early in December a small black spot passed quietly over the face of the sun. Reflected by means of a telescope, upon a white sheet, the sun appeared the size of a saucer and the spot (the planet Venus) about the dimensions of the end of a cedar pencil.

Astronomers, at the expense of their governments, were busy in different parts of the world, carefully noting the particulars of this event, to the end that the true parallax might be learned, and thus the exact distance of the sun be established; previous observations and methods having left us in a doubt included between ninety-two millions and ninety-five millions of miles, a matter of vast importance in the settlement of mooted questions of astronomy.

According to Prof. Langley, who also discovered a spot upon Venus during the transit, the delicacy of the instruments and the care necessary in their manipulation can be realized when the task may be compared "to placing a human hair at a distance of one hundred and eighty yards and trying to measure its thickness."

A repetition of the passing of Venus *directly* between the earth and the sun will occur just one hundred and twenty years hence, and then perhaps, astronomers will be in possession of instruments as far superior to those which are now in use, as ours are to the tools of the observers of 1769, and the savans of to-day will be looked back upon complacently as earnest strugglers after knowledge, although from the advanced lights of that day, as groping in the dark.

It may be so, but we *are* groping. Not content to hide their candle under a bushel, the people of the nineteenth century are using their gifts, and at the close of each year vast strides are recorded in the advancement of knowledge and power.

In the usual contemplation of the past, incident to the closing of the year, it would be instructive to look back upon the period of the last pair of transits of Venus, 1761 and 1769, and note the difference between the known resources of our race then and now. I shall not attempt any full specification of them, nor shall I compare the methods of that period with those of this, however interesting it might be. My end will be reached if a few hints shall furnish a pleasant and useful task to any or all the readers of the BISTOURY.

The newspapers throughout the United States, within *forty-eight hours*, published reports from remote parts of the world, New Zealand, Tasmania, etc., of the success of the astronomical observations of this last transit, at those points.

Photographs of the sun to the amount of three hundred different views, during the six hours of the transit were taken at one station.

The spectroscope also revealed secrets of Venus, and even determined, according to Prof. Young, of Princeton, the presence of water there; suggesting the probability of inhabitants.

Neither of the three agents in producing these results, were dreamed of in 1769. All of them are but in their infancy now.

It is but lately that the velocity of light has been accurately ascertained. Its use as a printing press dates back but to Daguerre, and even his plates have almost been forgotten in the wonderful improvements of photography. The writer of this remembers distinctly the first daguerreotypes ever taken in this country, they were by Dr. Paul B. Goddard, an earnest worker in science and a surgeon of great celebrity at that time in Philadelphia.

Now, the sun takes his own negatives from which any number of duplicates may be made by exposure to his rays. The same art that portrays the magnificent center of our system, also pictures the minutest objects of the microscope.

The mighty power of electricity, since the period of the last twin transits, has been discovered, and although not yet fully understood, the zeal and courage of the nineteenth century have brought it into uses of great value to mankind, and earnest brains are daily developing new plans for its further subjection and usefulness.

The contemplation of the different and wonderful uses of this force of nature under the discoveries of the century will furnish not only gratification but surprise.

Electricity is not only part of our physical life, but has become such a powerful agent in social and business affairs, that it is hard to realize that we ever could be without its services. Cities are lighted brilliantly by it; citizens are transported in cars propelled by it; space is almost annihilated through its agency. Money transfers are now made from one part of the world to another with as much facility and in as short a time as if the transactions were between merchants in the same city. Without the telegraph and telephone, how could the enormous traffic of our railroads be conducted with the speed and safety now reached?

Here naturally follow the wonderful developments of steam. We can not claim its discovery, for that dates back to Hero's little engine during the third century B. C. But since the last twin transits of Venus, steam has been made the servant of man in all things requiring power, and the almost incredible results of that service are subjects worthy of close consideration.

The spectrum analysis, although comparatively unknown except amongst scientists, is one of the most brilliant discoveries of the century. There is not space in an article like this, nor would it coincide with the intention of the writer, to enter into a full explanation of the spectroscope and its uses, the aim is only to give heads of subjects for investigation and thought. But amidst the wonders of science, not the least is a little prism which to the initiated reveals the component elements of worlds millions of miles away. This is not mere theory, its correctness can be easily proven by practical tests. Perhaps ere the next transit of Venus, science will have reached—where?

In the interim of 1769 and 1882, our great Republic sprang into existence, struggled through a brilliant childhood and reached the position of a centenarian, forcing itself into the wonder and respect of the governments of the world. Its development has not been a quiet one, but through fierce struggles with the parent country in its infancy, exciting party contests farther on, and even an internecine war to wipe from its escutcheon the blot of slavery, yet to-day it stands proudly before the world the peer of all nations.

Within itself, under the system of public education, in spite of the counter influences of superstitious bigotry, greed, and a large influx of a class emigrants too ignorant to comprehend the true principles of a republic, and used as tools for evil by unscrupulous men, the people are fast rising to the dignity of their position as self-governors, and are showing their determination to perpetuate our institutions by displacing those "who have been weighed in the balance and found wanting," and electing to office men known to be capable and honest. The closing of the year is certainly marked by a wonderful step forward in the political affairs of our country—an enormous surplus in the treasury, Congress hard at work trying to judiciously cut down the income, creditors only anxious because they fear the debt *will* be paid. Truly much have we as a nation to be thankful for and justifiably proud of.

With astronomy, science, patriotism and a little politics before them as suggestive of retrospection and prospection, I close my communications with the readers of the BISTOURY for 1882, wishing them and theirs a happy New Year.

